# Outcomes of *Stenotrophomonas maltophilia* hospital-acquired pneumonia in intensive care unit: a nationwide retrospective study

**DOI:** 10.1186/s13054-019-2649-5

**Published:** 2019-11-21

**Authors:** Philippe Guerci, Hugo Bellut, Mokhtar Mokhtari, Julie Gaudefroy, Nicolas Mongardon, Claire Charpentier, Guillaume Louis, Parvine Tashk, Clément Dubost, Stanislas Ledochowski, Antoine Kimmoun, Thomas Godet, Julien Pottecher, Jean-Marc Lalot, Emmanuel Novy, David Hajage, Adrien Bouglé, AZUREA research network, Jean-Michel Constantin, Thomas Godet, Philippe Guerci, Sebastien Perbet, Stanislas Ledochowski

**Affiliations:** 1 Department of Anaesthesiology and Critical Care Medicine, Institut Lorrain du Coeur et des Vaisseaux, University Hospital of Nancy-Brabois, Vandoeuvre-Lès-Nancy, France; 2 INSERM U1116, Groupe Choc, University of Lorraine, Nancy, France; 3 Sorbonne Université, Assistance Publique - Hôpitaux de Paris (AP-HP), Department of Anaesthesiology and Critical Care Medicine, Institute of Cardiology, Pitié-Salpêtrière Hospital, 47-83 Boulevard de l’Hôpital, 75013 Paris, France; 4 Service d’Anesthésie-Réanimation Chirurgicale, Hôpital Hautepierre, Hôpitaux Universitaires de Strasbourg, Strasbourg, France; 5 Service d’Anesthésie-Réanimation, Hôpital Henri Mondor, DMU CARE, Assistance Publique - Hôpitaux de Paris (AP-HP), Inserm U955 équipe 3, Université Paris-Est Créteil, Créteil, France; 6 Réanimation Chirurgicale Polyvalente, Hôpital Central, Centre Hospitalier Universitaire de Nancy, Nancy, France; 7 Réanimation polyvalente, Hôpital de Mercy, CHR Metz-Thionville, Metz, France; 8 Service d’Anesthésie-Réanimation, Hôpital Bichat-Claude Bernard, Assistance Publique - Hôpitaux de Paris (AP-HP), Paris, France; 9 Réanimation polyvalente, Hôpital d’Instruction des Armées (HIA) Bégin, Saint-Mandé, France; 10 Service de Réanimation Polyvalente, Groupement Hospitalier Nord Dauphiné- Centre Hospitalier Pierre Oudot, Bourgoin-Jallieu, France; 11 Réanimation Médicale, Institut Lorrain du Cœur et des Vaisseaux, CHU Nancy-Brabois, Vandoeuvre-Lès-Nancy, France; 12 Réanimation Adultes et Soins Continus, Pôle de Médecine Péri-opératoire, Hôpital Estaing, Centre Hospitalier Universitaire de Clermont-Ferrand, Clermont-Ferrand, France; 13 Faculté de Médecine, Institut de Physiologie, EA3072, Fédération de Médecine Translationnelle de Strasbourg (FMTS), Université de Strasbourg, Strasbourg, France; 14 Service d’Anesthésie-Réanimation, Réanimation polyvalente, Centre Hospitalier Emile Durkheim, Epinal, France; 15 Département Biostatistique Santé Publique Et Information Médicale, Unité de Recherche Clinique PSL-CFX, Centre de Pharmacoépidémiologie (Cephepi), Sorbonne Université, INSERM, Institut Pierre Louis de Santé Publique, Equipe Pharmacoépidémiologie et évaluation des soins, AP-HP, Hôpital Pitié-Salpêtrière, CIC-1421, Paris, France

**Keywords:** Hospital-acquired pneumonia, *Stenotrophomonas maltophilia*, Intensive care, Antimicrobial therapy, Combination therapy

## Abstract

**Background:**

There is little descriptive data on *Stenotrophomonas maltophilia* hospital-acquired pneumonia (HAP) in critically ill patients. The optimal modalities of antimicrobial therapy remain to be determined. Our objective was to describe the epidemiology and prognostic factors associated with *S. maltophilia* pneumonia, focusing on antimicrobial therapy.

**Methods:**

This nationwide retrospective study included all patients admitted to 25 French mixed intensive care units between 2012 and 2017 with hospital-acquired *S. maltophilia* HAP during intensive care unit stay. Primary endpoint was time to in-hospital death. Secondary endpoints included microbiologic effectiveness and antimicrobial therapeutic modalities such as delay to appropriate antimicrobial treatment, mono versus combination therapy, and duration of antimicrobial therapy.

**Results:**

Of the 282 patients included, 84% were intubated at *S. maltophilia* HAP diagnosis for duration of 11 [5–18] days. The Simplified Acute Physiology Score II was 47 [36–63], and the in-hospital mortality was 49.7%. Underlying chronic pulmonary comorbidities were present in 14.1% of cases. Empirical antimicrobial therapy was considered effective on *S. maltophilia* according to susceptibility patterns in only 30% of cases. Delay to appropriate antimicrobial treatment had, however, no significant impact on the primary endpoint. Survival analysis did not show any benefit from combination antimicrobial therapy (HR = 1.27, 95%CI [0.88; 1.83], *p* = 0.20) or prolonged antimicrobial therapy for more than 7 days (HR = 1.06, 95%CI [0.6; 1.86], *p* = 0.84). No differences were noted in in-hospital death irrespective of an appropriate and timely empiric antimicrobial therapy between mono- versus polymicrobial *S. maltophilia* HAP (*p* = 0.273). The duration of ventilation prior to *S. maltophilia* HAP diagnosis and ICU length of stay were shorter in patients with monomicrobial *S. maltophilia* HAP (*p* = 0.031 and *p* = 0.034 respectively).

**Conclusions:**

*S. maltophilia* HAP occurred in severe, long-stay intensive care patients who mainly required prolonged invasive ventilation. Empirical antimicrobial therapy was barely effective while antimicrobial treatment modalities had no significant impact on hospital survival.

**Trial registration:**

clinicaltrials.gov, NCT03506191

## Background

*Stenotrophomonas maltophilia* is one of the 10 most frequently isolated pathogens responsible for hospital-acquired pneumonias (HAPs) in intensive care unit (ICU) patients in western countries [1, 2], representing approximately 5% of positive pulmonary samples. Previous studies identified several risk factors for developing *S. maltophilia* HAP in critically ill patients, such as prolonged ICU hospitalization associated with invasive procedures, extended periods of mechanical ventilation, or exposure to broad-spectrum antibiotics [3–5]. Therefore, *S. maltophilia* pneumonia occurs preferentially in patients with the poorest prognosis [6, 7]. However, these studies were conducted from heterogeneous and small cohorts of patients. The severity of *S. maltophilia* HAP and antimicrobial therapy modalities were sparsely reported [3, 4]. Hence, data are lacking to draw recommendations on the optimal therapeutic strategies against *S. maltophilia* pneumonia.

We undertook a large nationwide multicenter retrospective study with the main objective to demonstrate that modalities of antibiotic therapy, including empirical antimicrobial choice, whether a combination therapy was used, or the duration of the therapy, would influence in-hospital mortality. Secondary objectives were to describe the characteristics of ICU patients with *S. maltophilia* HAP and to draw prognostic factors of these pneumonias.

## Methods

### Design of the study and setting

The medical records of patients who experienced *S. maltophilia* pneumonia from January 2012 to January 2017 were collected from 25 ICUs of the French Society of Anaesthesia & Intensive Care Medicine (SFAR) and AZUREA networks [8]. Participating centers and case-mixes are listed in Additional file 1.

The collected data involved both ICU and hospital stays. Follow-up was stopped either after hospital discharge or death, whichever occurred first.

### Participants

#### Eligibility criteria

All patients aged over 18 years who were admitted to the participating ICUs and presenting with a documented diagnosis of *S. maltophilia* pneumonia during their ICU stay were eligible.

#### Source and method of selection

The patient’s files were extracted through French hospital discharge database containing individual records of all hospital stays using International Classification of Disease (ICD-10) for the terms “*Stenotrophomonas maltophilia*” and “pneumonia.” In addition, ICU medical charts were cross-checked with microbiology laboratory-specific information systems to ensure exhaustivity.

Each medical record was analyzed by local investigators to determine if clinical, biological, and/or radiological signs of *S. maltophilia* HAP were present, thus excluding respiratory tract colonizations (defined as a positive respiratory sample without clinical, biological, and/or radiological signs of *S. maltophilia* pneumonia). In case of uncertainty, consensus was obtained between local infectious disease specialists and study coordinators (PG, AB) to clarify *S. maltophilia* HAP cases.

#### Definitions

Pneumonia was defined as follows: (i) new or progressive lung infiltrate, (ii) temperature > 38 °C or < 36.5 °C, leukocyte count > 12,000 μl^−1^ or < 4000 μl^−1^, purulent endotracheal aspirate or sputum, (iii) positive respiratory sample (see below), and (iv) decline in oxygenation [9, 10]. HAP was defined as a pneumonia not incubating at the time of hospital admission and occurring 48 h or more after admission. Ventilator-associated pneumonia (VAP) was defined as a pneumonia occurring 48 h or more after tracheal intubation [9].

The clinical cure of *S. maltophilia* pneumonia was defined by the absence of pneumonia criteria 48 h after antimicrobial therapy cessation. Treatment failure was defined as a failure of first-line treatment or death attributable to *S. maltophilia* pneumonia. Recurrence was defined as the onset of new pneumonia criteria associated with a positive respiratory sample with *S. maltophilia* after the initial pneumonia was considered successfully cured.

Empirical antimicrobial therapy was defined as the first agents prescribed for the initial treatment of HAP (effective or not on *S. maltophilia*) finally diagnosed as being caused by *S. maltophilia*. Empirical antimicrobial therapy was considered as effective if the *S. maltophilia* strain cultured from the respiratory sample was susceptible to at least one of the antimicrobial agents. Combination therapy was defined as the administration of at least two antimicrobial agents a priori (before *S. maltophilia* HAP has been confirmed, usually within 48 h) or a posteriori (after *S. maltophilia* HAP has been confirmed) effective on the *S. maltophilia* strain for more than 24 h.

#### Data collection

Usual demographic variables were collected, including previous hospital stays and previous exposure to antimicrobial therapies (agents and durations). Simplified Acute Physiology Score II (SAPSII) and the Sequential Organ Failure Assessment (SOFA) score were assessed.

On the day of *S. maltophilia* HAP diagnosis, the SOFA score was collected, as well as the number and type of invasive devices inserted. The severity of hypoxemia was graded according to the Berlin acute respiratory distress syndrome (ARDS) criteria [11]. Requirements for high-flow nasal oxygen therapy, non-invasive or invasive mechanical ventilation, or extracorporeal membrane oxygenation (ECMO) were reported. Empirical antimicrobial therapy and secondary adaptations were recorded, as were durations.

#### Diagnosis of positive bacterial culture

In case of *S. maltophilia* isolation, the culture was considered to be positive (either mono- or polymicrobial infection) with the following cutoff: (1) for minimally contaminated lower respiratory tract sample with quantitative culture, the threshold was 10^4^ colony-forming units (CFU)/ml for bronchoalveolar lavage (BAL) and the cutoff was 10^3^ CFU/ml for protected specimen brush (PSB) or protected (plugged) telescoping catheter (PTC); (2) nonprotected sample (endotracheal aspirate, ETA) with quantitative culture (10^5^ CFU/ml); or (3) sputum bacteriology with quantitative culture (10^7^ CFU/ml) [12].

#### Antimicrobial susceptibility testing (AST)

*S. maltophilia* identification characteristics (date of isolation and type of respiratory tract sampling) and antimicrobial susceptibility testing were independently performed by each microbiology laboratory. AST was performed on isolates using disk diffusion or automated testing methods according to guidelines and breakpoints established by the European Committee on Antimicrobial Susceptibility Testing (EUCAST) [13].

### Data management

Data were collected and managed using Research Electronic Data Capture (REDCap) software [14]. The database was approved by the institutional review board of the SFAR (IRB00010254-2015-010), which waived the need for signed informed consent of the participants, in accordance with the French legislation on noninterventional studies [15]. The study was declared on clinicaltrials.gov (NCT03506191). This manuscript was written in accordance with the STROBE statement for the reporting of observational studies in epidemiology.

### Statistical analysis

The results are expressed as the number of patients (%) for categorical variables and mean (± standard deviation) or median [IQR] for continuous variables.

Prognostic factors associated with time to in-hospital death were studied using the Cox proportional hazard model. Time to in-hospital death was calculated from the diagnostic date of *S. maltophilia* to death. The follow-up was censored at discharge from the ICU and/or the hospital. Baseline prognostic factors were age, SAPS II, mechanical ventilation at diagnosis, VAP, duration of mechanical ventilation before the diagnosis, SOFA score at diagnosis, bacteremia, mono/polymicrobial pneumonia, use of empirical antimicrobial therapy, and use of empirical antimicrobial therapy effective against *S. maltophilia.* Other antimicrobial therapy-related variables were not defined as baseline and were thus entered in the model as time-dependent variables, including time elapsed between sample and effective antimicrobial therapy, use of effective combination antimicrobial therapy, and duration of effective antimicrobial therapy against *S. maltophilia* (monotherapy or combination therapy).

Baseline and time-dependent variables associated (*p* < 0.05) with outcome in the univariate analysis and that were present at the diagnosis were considered for the multivariate model, and the final model was selected using backward stepwise regression (*p* < 0.05). Hazard ratios (HR) were calculated accordingly with their 95% confidence intervals (CI).

We compared the time to in-hospital death between patients who received or not an empirical antimicrobial therapy effective against *S. maltophilia* using propensity score framework. The variables used for propensity score estimations were age, sex, SOFA score at diagnosis, SAPS II, and the ICU length of stay before pneumonia diagnosis. The two groups of patients were matched using a 1:1 nearest neighbor matching algorithm with replacement, using a caliper of 0.2 of the standard deviation of the propensity score on the logit scale [16]. Covariate balance between the two groups was assessed after matching, and we considered an absolute standardized difference (ASD) less than 0.1 as evidence of balance [17]. Then, time to in-hospital death was compared between matched groups using a Cox proportional hazard model. The 95% confidence intervals of the estimated hazard ratio (empirical antimicrobial therapy yes vs no) were estimated using robust standard error [18].

Significance was defined as *p* values < 0.05. Statistical tests were two-sided. Statistical analyses were performed using R 3.5.0 (R Foundation for Statistical Computing, http://www.R-project.org/, Vienna, Austria).

## Results

### Population

**Fig. 1:**
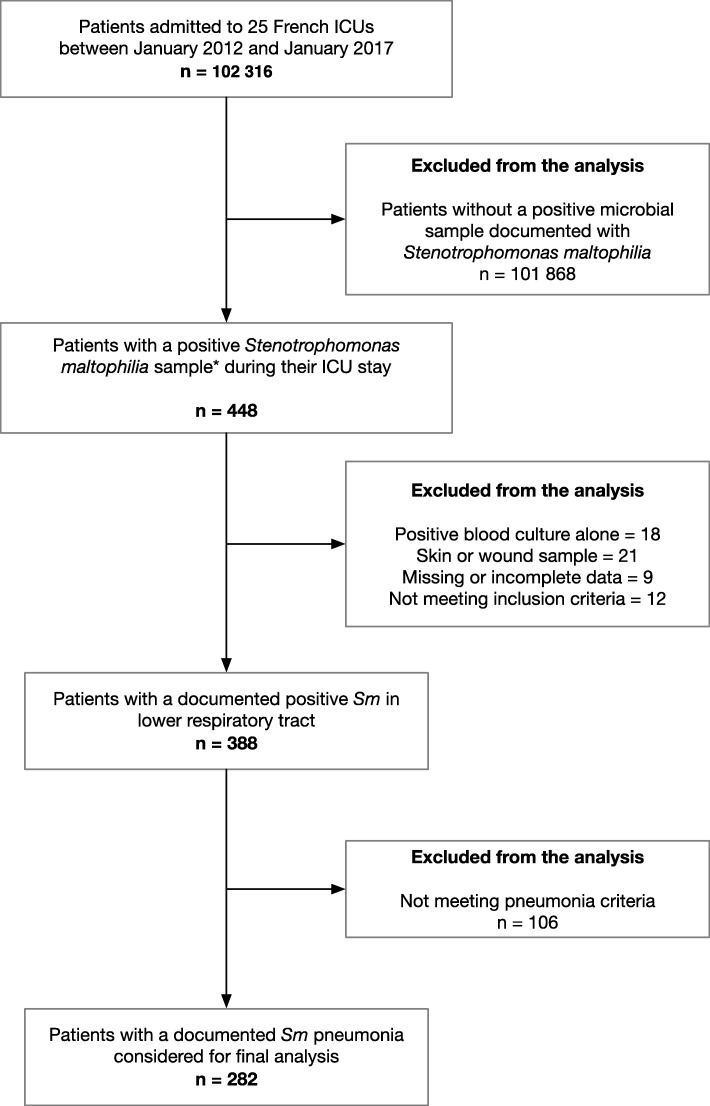
Flowchart of the inclusion of patients presenting with *Stenotrophomonas maltophilia* hospital-acquired pneumonia. Asterisk indicates that sample can be from lower respiratory tract, blood, wound/skin, or urine

Of the 102,316 patients admitted to the 25 ICUs within the study period, 282 (0.27%) with a *S. maltophilia* pneumonia were included (Fig. 1). Our population was predominantly male (69.9%), with an age of 65 [56–74] years, mostly admitted for medical reasons (59.2%) or emergent surgery (29.4%). Severity at admission was illustrated by SOFA score (8 [5–11]) and SAPSII (47 [36–63]). ICU and hospital lengths of stay were 32 [19–58] and 54 [30–94] days, respectively. The overall in-hospital mortality rate was 49.7% (Table 1). There was no difference in trends of patient inclusion and distribution over years. 48, 51, 60, 65, and 59 patients were included in 2012, 2013, 2014, 2015, and 2016 respectively with similar distributions in terms of mortality.

**Table 1:** Demographic and baseline characteristics of ICU patients with *Stenotrophomonas maltophilia* hospital-acquired pneumonia

Variables	Total *N* = 282
Gender, male	197 (69.9)
Age, years	65 [56–74]
BMI (kg m^− 2^)	25.3 [22–28.8]
Reason for ICU admission	
Medical condition	167 (59.2)
Scheduled surgery	32 (11.3)
Emergent surgery	83 (29.4)
Postoperative period of cardiac surgery	60 (21.3)
Previous carriage of *S. maltophilia*	13 (4.6)
Pulmonary comorbidities	
Chronic obstructive pulmonary disease	51 (18.1)
Chronic respiratory insufficiency	30 (10.6)
Cystic fibrosis	2 (0.7)
Other comorbidities	
Hypertension	147 (52.1)
Congestive heart failure	66 (23.4)
Dialysis-dependent chronic kidney disease	8 (2.8)
Liver cirrhosis	22 (7.8)
Insulin-requiring diabetes	31 (11)
Severe neurologic disability	30 (10.6)
Habits	
Active smoking	86 (30.5)
Chronic alcohol abuse	47 (16.7)
Drug abuse	8 (2.8)
Immune comprise conditions (*n*, %)	
Neutropenia	1 (0.3)
HIV and/or CD4 count < 50/mm^3^	1 (0.3)
Recent or ongoing chemotherapy	11 (3.9)
Hematologic malignancy	16 (5.7)
Solid tumor being actively treated	20 (7.1)
Solid tumor in remission	22 (7.8)
Immunosuppressive therapy or corticosteroids use > 0.5 mg/kg > 30 days	34 (12)
Innate or acquired immune deficiency	1 (0.3)
Severity scores	
SOFA score at admission	8 [5–11]
SAPS II at 24 h	47 [36–63]
Ventilator-associated pneumonia	228 (80.8)
Duration of ventilation before *S. maltophilia* HAP, days	11 [5–18]
ICU length of stay, days	32 [19–58]
Hospital length of stay, days	54 [30–94]
Number of days between hospital admission and *S. maltophilia* HAP	16 [8–27]
Number of days between ICU admission and *S. maltophilia* HAP	11 [5–19]
Mortality	140 (49.7)
Mortality directly attributable to *S. maltophilia*	34 (24.3)

Data are expressed as number and percentage or median [interquartile range] as appropriate

*BMI* body mass index, *HAP* hospital-acquired pneumonia, *HIV* human immunodeficiency virus, *ICU* intensive care unit, *IQR* interquartile range, *SAPS* Simplified Acute Physiology Score, *SD* standard deviation, *SOFA* Sequential Organ Failure Assessment, *S. maltophilia Stenotrophomonas maltophilia*

Patients had been hospitalized in the ICU for 11 [5–19] days at the time of onset of *S. maltophilia* pneumonia. Forty patients (14.2%) presented with a chronic underlying pulmonary disease. Other characteristics of patients are described in Table 1, and invasive devices are reported in Additional file 2: Table S1 in the online data supplement.

### Description of *Stenotrophomonas maltophilia* hospital-acquired pneumonia

Characteristics of *S. maltophilia* HAP are described in Table 2. Briefly, 41.6% of *S. maltophilia* pneumonias were monomicrobial and 80.8% were VAP. Blood culture was concomitantly positive in only 7.1% of cases.

**Table 2:** Characteristics of *Stenotrophomonas maltophilia* hospital-acquired pneumonia

Variables	Total *N* = 282
Monomicrobial pneumonia (*S. maltophilia* only) (*n*, (%))	117 (41.6)
Other microorganisms identified (polymicrobial) (*n*, (%))	
*Acinetobacter baumanii*	7 (4.3)
*Citrobacter* spp.	7 (4.3)
*Enterobacter* spp.	30 (18.3)
*Enterococcus* spp.	6 (3.7)
*Escherichia coli*	25 (15.2)
*Haemophilus*	3 (1.8)
*Hafnia alvei*	3 (1.8)
*Klebsiella* spp.	25 (15.2)
Methicillin-resistant *Staphylococcus aureus*	3 (1.8)
Methicillin-sensitive *Staphylococcus aureus*	13 (7.9)
*Morganella morganii*	1 (0.6)
*Proteus* spp.	7 (4.3)
*Pseudomonas aeruginosa*	49 (29.9)
*Serratia* spp.	8 (4.9)
*Streptococcus* spp.	1 (0.6)
Others	10 (6.1)
*S. maltophilia* pneumonia-related manifestations (*n*, (%))	
Associated bacteremia	20 (7.1)
Associated empyema thoracis	25 (8.9)
Associated septic shock	123 (43.6)
Total number of days of norepinephrine infusion (median [IQR])	0 [0–6]
Severity of pneumonia	
Oxygenation level regarding *S. maltophilia* HAP (*n*, (%))	
Hypoxemia with PaO_2_/FIO_2_ > 300 mmHg with PEEP ≥ 5 cmH_2_O	45 (16)
Mild ARDS	98 (34.7)
Moderate ARDS	101 (35.8)
Severe ARDS	36 (12.7)
Prone positioning (*n*, (%))	13 (4.6)
ECMO requirement	15 (5.3)
Veno-venous	8 (53.3)
Veno-arterial	7 (46.7)

Data is presented as number (%) or median [IQR]. Mild ARDS (200 mmHg < PaO_2_/FiO_2_ ≤ 300 mmHg with PEEP ≥ 5 cmH_2_O), moderate ARDS (100 mmHg < PaO_2_/FiO_2_ ≤ 200 mmHg with PEEP ≥ 5 cmH_2_O), severe ARDS (PaO_2_/FiO_2_ ≤ 100 mmHg with PEEP ≥ 5 cmH_2_O)

*S. maltophilia*, *Stenotrophomonas maltophilia*; *ARDS*, Acute Respiratory Distress Syndrome, was defined according to the Berlin definition; *PEEP*, positive end-expiratory pressure

Microbiological diagnosis methods for isolation of *S. maltophilia* are presented in Additional file 3: Table S2 in the online data supplement. Patients with *S. maltophilia* VAP had a duration of mechanical ventilation before the onset of pneumonia of 11 [5–18] days. *S. maltophilia* pneumonia-related septic shock was present in 123 patients (43.6%) within 48 h of *S. maltophilia* HAP (septic shock attributed to pneumonia by clinicians and without other identified cause in the post hoc analysis). Among these patients who developed septic shock, 38 (30.8%) did not receive initial empirical antimicrobial therapy. Forty-nine percent of patients fulfilled moderate or severe ARDS criteria.

### Antimicrobial therapy

The description of antimicrobial therapy modalities is reported in Table 3. Before the onset of *S. maltophilia* pneumonia, patients received 3 [2–4] prior antimicrobial therapies for at least 5 consecutive days in ICU. Empirical antimicrobial therapy was a posteriori effective against *S. maltophilia* in 30.1% of cases. The duration of effective antimicrobial therapy on *S. maltophilia* was 11 [7–15] days. A combination of antimicrobials effective against *S. maltophilia* was used in 59.4% of patients for 7 [5–12] days.

**Table 3:** Antimicrobial therapy management related to *Stenotrophomonas maltophilia* hospital-acquired pneumonia

Variables	Total *N* = 282
Number of antimicrobial therapy lines administered within 30 days before diagnosis (median [IQR])	3 [2–4]
Number of days with initial ineffective antimicrobial therapy on *S. maltophilia* (median [IQR])	2 [2–3.5]
Most commonly prescribed antimicrobial agents before *S. maltophilia* HAP onset (*n*, (%))	
Amoxicillin	20 (7.1)
Amoxicillin–clavulanate	61 (21.6)
Third-generation cephalosporin	93 (33)
Cefepime	25 (8.9)
Ceftazidime	17 (6)
Ticarcillin	4 (1.4)
Ticarcillin–clavulanate	10 (3.5)
Piperacillin	15 (5.3)
Piperacillin–tazobactam	97 (34.4)
Carbapenem	63 (22.3)
Aminoglycoside	70 (24.8)
Fluoroquinolone	40 (14.2)
Trimethoprim–sulfamethoxazole	9 (3.2)
Glycopeptide	59 (20.9)
Metronidazole	32 (11.3)
Linezolid	31 (11)
Others	71 (25.2)
Empirical antimicrobial therapy (*n*, (%))	166 (58.8)
Number of antimicrobial agents for empirical antimicrobial therapy (median, [IQR])	1 [0–2]
Efficient empirical therapy on *S. maltophilia* (*n*, (%))	50 (30.1)
Combination antimicrobial therapy (2 or more) targeting *S. maltophilia* (*n*, (%))	167 (59.4)
Duration of antimicrobial therapy targeting *S. maltophilia*, days (median, [IQR])	11 [7–15]
Duration of combined (2 or more) antimicrobial therapy targeting *S. maltophilia*, days (median, [IQR])	7 [5–12]

*S. maltophilia* is intrinsically resistant to amoxicillin, amoxicillin/clavulanate, ticarcillin, piperacillin/tazobactam, carbapenems, and aminoglycosides (EUCAST expert rules version 3.1—26 Sept 2016). Data are presented as median, interquartile range ([IQR]), or number (percentage) (*n*, (%)) as appropriate

*HAP* hospital-acquired pneumonia

### Microbiological data

**Fig. 2:**
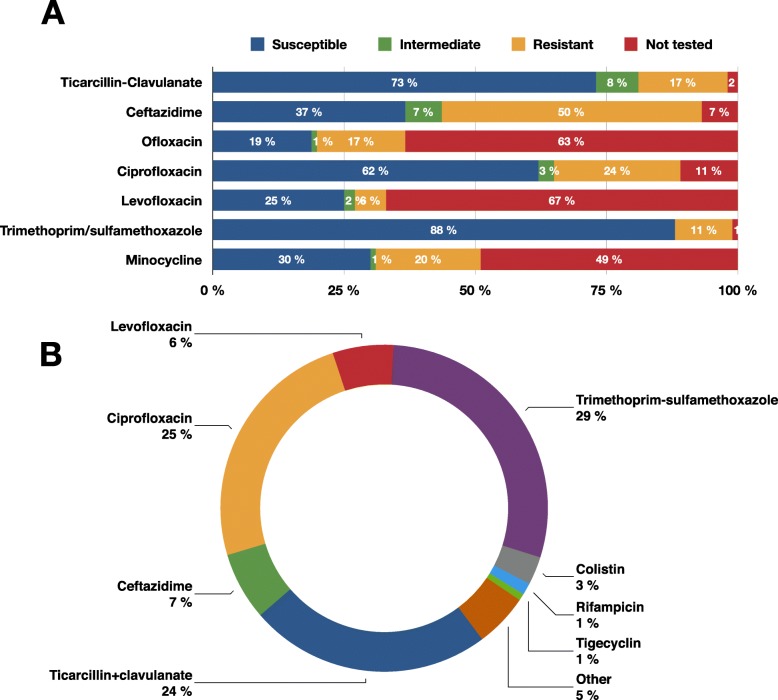
**a** Antibiotic susceptibility of *Stenotrophomonas maltophilia* strains isolated from the respiratory tract samples (*n* = 282). **b** Efficient antibiotic treatments prescribed to treat *Stenotrophomonas maltophilia* hospital-acquired pneumonia. Antibiotic susceptibility is depicted in percentage (%) of isolates that were susceptible, intermediate, and resistant or when the antibiotic treatment was not assayed

AST of *S. maltophilia* strains is presented in Fig. 2a. Trimethoprim–sulfamethoxazole (TMP-SMX) (88.1%) and ticarcillin–clavulanate (73.3%) remained highly active against more than two thirds of *S. maltophilia* strains. The main antimicrobial therapies prescribed to treat *S. maltophilia* HAP after identification were TMP-SMX (29%), ciprofloxacin (25%), and ticarcillin–clavulanate (24%) (Fig. 2b).

### Prognosis

Treatment failure occurred in 65 patients (23.1%). Recurrence of *S. maltophilia* pneumonia was diagnosed in 48 patients (17.0%). Co-infection with another pathogen, mostly *Pseudomonas aeruginosa* (*n* = 30), was diagnosed in 70 patients (24.8%) during recurrence (Additional file 4: Table S3).

In univariate analysis, variables associated with the primary endpoint (i.e., time to in-hospital death) were age (HR = 1.025, 95%CI [1.012; 1.038], *p* = 0.0001), SAPS II (HR = 1.009, 95%CI [1.001; 1.018], *p* = 0.036), and SOFA score at pneumonia diagnosis (HR = 1.099, 95% CI [1.057; 1.142], *p* < 0.0001) (Table 4). The subsequent occurrence of a septic shock was significantly associated with an increased risk of death (HR = 3.070, 95%CI [1.9; 5.0], *p* < 0.0001).

**Table 4:** Variables associated with the time to in-hospital death

Variables	Univariate analysis		Multivariate analysis	
	HR [95%CI]	*p* value	HR [95%CI]	*p* value
Age	1.025 [1.012; 1.038]	0.0001	1.02 [1.01; 1.04]	0.001
SAPS II	1.009 [1.001; 1.018]	0.036		
Mechanical ventilation at diagnosis	1.692 [0.83; 3.46]	0.151		
VAP	0.748 [0.49; 1.14]	0.178		
Duration of MV before the diagnosis	0.997 [0.988; 1.006]	0.462		
SOFA score at diagnosis	1.099 [1.057; 1.142]	< 0.0001	1.1 [1.06; 1.15]	< 0.001
Bacteremia	0.814 [0.41; 1.60]	0.551		
Monomicrobial pneumonia	1.368 [0.98; 1.91]	0.066		
Co-infection with *Pseudomonas aeruginosa*	0.843 [0.53; 1.33]	0.464		
Time elapsed between pulmonary sample and effective antimicrobial therapy	0.99 [0.97; 1.01]	0.434		
< 24 h	1			
24–48 h	1.22 [0.74; 2]	0.429		
> 48 h	1.34 [0.85; 2.12]	0.204		
Empirical antibiotic therapy	1.019 [0.73; 1.43]	0.914		
Subsequent septic shock	3.070 [1.9; 5.0]	< 0.0001		
Empirical antibiotic therapy effective against *S. maltophilia*	0.839 [0.52; 1.35]	0.4705		
Effective combination antimicrobial therapy	1.27 [0.88; 1.83]	0.204		
Duration of effective antimicrobial therapy against *S. maltophilia*	1.03 [0.98;1.07]	0.243		
< 7 days	1			
7–14 days	1.06 [0.6; 1.86]	0.842		
> 14 days	0.88 [0.45; 1.71]	0.706		
Duration of effective combination therapy against *S. maltophilia*	0.99 [0.96; 1.03]	0.679		
< 7 days	1			
7–14 days	0.82 [0.52; 1.29]	0.393		
> 14 days	0.71 [0.39; 1.29]	0.262		

*SAPS* Simplified Acute Physiology Score, *VAP* ventilator-associated pneumonia, *MV* mechanical ventilation, *HR* hazard ratio, *S. maltophilia Stenotrophomonas maltophilia*, *SOFA* Sequential Organ Failure Assessment

Neither the duration of treatment nor the use of combination therapy directed against *S. maltophilia* was associated with the primary endpoint (Table 4). Other commonly reported risk factors for *S. maltophilia* HAP (i.e., immunosuppression, chronic obstructive pulmonary disease (COPD), prior antimicrobial therapy) were not statistically associated with time to in-hospital death.

In multivariate analysis, only age (HR = 1.02, 95% CI [1.01; 1.04], *p* = 0.001) and SOFA score at *S. maltophilia* pneumonia diagnosis (HR = 1.1, 95%CI [1.06; 1.15], *p* < 0.001) were associated with in-hospital death (Table 4). Subsequent septic shock was not included in the multivariate analysis because it was a consequence and not present at the diagnosis of *S. maltophilia*. The results were similar when only considering VAP in our cohort (*N* = 228) (Additional file 5: Table S4). Finally, we performed a statistical analysis based on a propensity score to evaluate the effect of an empirical antibiotic therapy effective on *S. maltophilia* on the primary endpoint (Additional file 6: Table S5). After matching, we compared 48 patients who received appropriate empirical antibiotic therapy versus 222 who did not (Additional file 7: Table S6). This analysis confirmed the previous results (HR = 0.891, 95%CI [0.498–1.593], *p* = 0.697).

### Mono- and polymicrobial *S. maltophilia* HAP

The aforementioned results remained unchanged when considering only monomicrobial *S. maltophilia* pneumonia (*n* = 117) (HR = 1.08, 95%CI [1.01; 1.15], *p* = 0.021 for SOFA score at *S. maltophilia* pneumonia diagnosis). Comparisons of characteristics and outcomes between mono- and polymicrobial *S. maltophilia* HAP are provided in Table 5. No differences were noted in in-hospital death irrespective of an appropriate and timely empiric antimicrobial therapy between mono- versus polymicrobial *S. maltophilia* HAP. A similar number of VAP occurred in both groups, 91 (77.8%) versus 136 (82.9%) (*p* = 0.280) for mono- and polymicrobial HAP respectively. The duration of ventilation prior to *S. maltophilia* HAP diagnosis and ICU length of stay were shorter in patients with monomicrobial *S. maltophilia* HAP.

**Table 5:** Characteristics and outcomes comparing patients with mono- versus polymicrobial *Stenotrophomonas maltophilia* HAPs

Variables	*Sm* only *N* = 117	Polymicrobial *Sm* HAP *N* = 164	*p* value
Gender, male	78 (66.7)	119 (72.6)	0.362
Age, years	67 [59–76]	64 [55–72]	*0.024*
BMI (kg m^−2^)	24.6 [21.1–29.6]	25.7 [22.3–28.4]	0.361
Reason for ICU admission			*0.024*
Medical condition	80 (68.4)	87 (53)	
Scheduled surgery	8 (6.8)	24 (14.6)	
Emergent surgery	29 (24.8)	53 (32.3)	
Previous carriage of *S. maltophilia*	5 (4.3)	6 (3.7)	0.812
Pulmonary comorbidities			
Chronic obstructive pulmonary disease	27 (23.1)	24 (14.6)	0.084
Chronic respiratory insufficiency	15 (12.8)	14 (8.5)	0.245
Cystic fibrosis	0 (0)	2 (1.2)	0.512
Prior exposure to carbapenems	27 (23.1)	37 (22.5)	0.723
Susceptibility to trimethoprim–sulfamethoxazole	97 (82.9)	147 (89.6)	0.424
Severity scores			
SOFA score at admission	8 [5–11]	8 [5–11]	0.381
SAPS II at 24 h	48 [37–62]	47 [36–63]	0.672
Ventilator-associated pneumonia	91 (77.8)	136 (82.9)	0.280
Duration of mechanical ventilation prior to *S. maltophilia* HAP, days	9 [2–17]	11 [6–20]	*0.031*
ICU length of stay, days	28 [16–49]	36 [22–60]	*0.034*
Hospital length of stay, days	45 [26–80]	59 [32–98]	0.062
Number of days between hospital admission and *S. maltophilia* HAP	14 [8–23]	17 [8–31]	0.294
Number of days between ICU admission and *S. maltophilia* HAP	10 [5–18]	12 [6–20]	*0.046*
Empiric antibiotic therapy	67 (57.3)	99 (60.4)	0.602
Combination therapy active on *S. maltophilia*	66 (56.4)	100 (61.0)	0.407
Overall in-hospital mortality	63 (53.8)	74 (45.1)	0.228
In-hospital mortality according to empiric antimicrobial therapy			0.273
Appropriate	8 (6.8)	16 (9.8)	
Inappropriate	24 (20.5)	30 (18.3)	

Data are presented as median, interquartile range ([IQR]), or number (percentage) (*n*, (%)) as appropriate

*BMI* body mass index, *HAP* hospital-acquired pneumonia, *SAPS* Simplified Acute Physiology Score, *SOFA* Sequential Organ Failure Assessment, *S. maltophilia Stenotrophomonas maltophilia*

## Discussion

Herein, we report the largest cohort study of critically ill patients developing *S. maltophilia* HAP. Regarding the large screening, the prevalence of *S. maltophilia* HAP remained very low. The majority of *S. maltophilia* HAP was VAP and occurred in patients ventilated for more than 10 days and previously exposed to several antimicrobial therapies. The mortality rate of these patients remained high, but surprisingly, the treatment delay in adequate antimicrobial therapy targeting *S. maltophilia* was not found to be associated with mortality. This observation may be the result of (i) a low virulence of the pathogen, (ii) the underlying condition of the critically ill patient being more contributive to the outcome than *S. maltophilia* HAP itself, or (iii) a 24- to 48-h delay in the treatment of *S. maltophilia* HAP had no real impact. Finally, if SAPSII and SOFA score were independently associated with mortality, no specific pneumonia or antimicrobial therapy-related factors impacted the outcome.

Our study population shares common features with previously published reports [3, 4, 6, 7, 19–23]. Indeed, *S. maltophilia* pneumonia develops in high-risk phenotypes patients, i.e., long ICU/hospital length of stay and duration of mechanical ventilation. Despite a mortality rate of approximately 50%, it is difficult to delineate direct attributable mortality of *S*. *maltophilia* HAP from mortality linked to underlying diseases [24]. Indeed, the prolonged ICU length of stay preceding *S*. *maltophilia* isolation and the number of prior antimicrobial therapies suggest noticeable patient frailty and complicated medical history. These factors have been associated to high mortality in patients with resistant bacteria in ICU [25]. *S*. *maltophilia* HAP could also be perceived as a final septic insult in long-stayer patients, promoting care withdrawal from the medical team.

Previous studies that included a small number of patients [3, 4, 6, 19–23, 26–31] suggested that immune-compromised conditions, COPD, prior cardiac surgery, or prior antimicrobial therapy were risk factors for *S. maltophilia* HAP. Conversely, our large series of mixed ICU patients did not confirm these elements. This implies that the involvement of *S. maltophilia* in late onset HAP should be considered and be kept in mind by all critical care physicians. However, in our series, initial antimicrobial therapy inactive against *S. maltophilia* was not a risk factor for in-hospital mortality, arguing against a systematic coverage of *S. maltophilia* by empirical antimicrobial therapy in this setting.

Although the prolonged duration of antimicrobial treatment is a well-known risk factor for emergence of multidrug resistant (MDR) bacteria [32], it did not appear to be discriminant in our study, irrespective of the class of antimicrobial agent previously administered. These results are in accordance with previously published literature on continuation or de-escalation of beta-lactam antibiotics and emergence of MDR [31]. Indeed, different regimens were used in our population, with various durations of treatment before *S. maltophilia* HAP diagnosis without apparent consequences on *S. maltophilia* emergence and susceptibility profiles. Soubirou et al. found that the increase in use of antimicrobial class was an independent predictor of *S. maltophilia* emergence in VAP [33]. It is however conceivable that some patients of our cohort may be colonized with other non-fermenting Gram-negative bacilli. Actually, almost 20% of patients had COPD or chronic respiratory insufficiency and might be regularly exposed to antibiotics. Nseir et al. and Saugel et al. reported 63% and 25% respectively incidence of COPD patients with *S. maltophilia* pneumonia [3, 4].

Despite clinical signs of HAP, only 59% of patients readily received empirical antimicrobial therapy (Additional file 4: Table S3). This highlights the variable implementation and adherence to antimicrobial bundles of care and stewardship programs [34–36]. Pathmanathan et al. previously reported no measurable impact of antibiotic therapy in patients without evidence of consolidation which suggests colonization [23]. In our study, colonization was excluded. Although it is currently suggested to start antibiotics early in patients with suspected VAP [37], physicians may have been expecting a definitive identification with the resistance profile of microorganisms possibly involved to restrain the use of broad-spectrum antibiotics, especially in patients previously exposed to several antibiotic regimens. Tracheobronchitis and pneumonia may also be hard to be differentiated and need time to be distinguished.

Of note, the interplay between resistance and virulence remains complex [38]. In these patients already exposed to several series of antimicrobial therapies, with an extended hospital length of stay, the likelihood of MDR bacteria involvement was very high. Moreover, due to its natural resistance to multiple antimicrobial agents, only one third of empirical antimicrobial therapies was actually effective against *S. maltophilia.* Conversely to other authors [39], but in accordance with studies on *Pseudomonas aeruginosa* VAP, the delayed administration of effective antimicrobial treatment was not statistically associated with increased mortality [40, 41].

The duration of *S. maltophilia* HAP antimicrobial therapy is still subject to debate. The comparison of short (< 8 days) versus prolonged (8 days and greater) antibiotic course could not properly be investigated in our study because of its design. Chastre et al. demonstrated that an 8-day course of antibiotic therapy for VAP was not inferior to a longer duration, but only 0.8% of patients had an *S. maltophilia* VAP [42]. However, it was suggested that patients infected with difficult-to-treat pathogens, immunocompromised patients, and patients at high risk for relapse may require a longer duration of antibiotic therapy. In our study, we identified neither the duration of antimicrobial treatment nor the combination of antibiotic therapies as significant risk factors for in-hospital death. Low virulence of *S. maltophilia* strains may partially explain these findings. In a recent retrospective study focused on the interest of combination therapy, Shah et al. reported that combination of antibiotic therapies yielded similar clinical efficacy and resistance development compared to monotherapy [43].

Optimal antimicrobial against *S. maltophilia* HAP may raise some concerns. The *S. maltophilia* strains in our study had a preserved susceptibility to ticarcillin–clavulanate and TMP-SMX, 73 and 88% respectively as expected [44]. However, only 29% of *S. maltophilia* HAP were treated with TMP-SMX. These discrepancies may be related to ICU physicians’ habits, the fear of TMP-SMX-related side effects, and the type of antibiotics administered prior to occurrence of *S. maltophilia* HAP. The use of fluoroquinolones could have been considered easier. When prescribed, TMP-SMX was combined with another antibiotic effective on *S. maltophilia* in 80% of cases. In addition, antimicrobial agent shortages change antimicrobial therapy armamentarium, with a cessation of manufacture of ticarcillin–clavulanate in 2015 [45]. Although ceftazidime/avibactam has poor activity on *S. maltophilia* [46], it may restore the susceptibility to aztreonam through the inhibition of the L2 β-lactamase in vitro [47]. The clinical efficacy of this combination has been reported in a case report of a transplant renal patient [48].

Augmented renal clearance can alter the pharmacokinetics and pharmacodynamics (PK/PD) of several antimicrobial agents, mainly ß-lactams [49]. The detailed dosages of antimicrobial agents and the measured creatinine clearance were not collected in the present study. However, TMP-SMX and fluoroquinolones were the most prescribed agents. Their plasma concentrations are not dramatically influenced by augmented renal clearance and not easily monitored in daily practice.

### Limitations

Our study differs from previous studies, where patients with and without *S. maltophilia* infection were compared. We did not consider *S. maltophilia* colonizations but only HAP, unlike previous studies [4]. In the case of a polymicrobial sample, it is uncertain which bacteria were responsible for the HAP. One may argue that our study suffers from inaccurate diagnoses differentiating between VAP and ventilator-associated tracheobronchitis due to its retrospective design using ICD codes. We acknowledge that the diagnosis method (ETA versus BAL or PSB) may influence the detection of *S. maltophilia*. This was a pragmatic study that describes different ICU practices, and to date, there is no formal evidence of improved outcomes depending on the diagnosis method used [50]. Despite strict inclusion criteria, and search for consensus in case of debatable case, it is possible that the physician’s judgment and diagnosis reported in the medical record were inaccurate. However, 80% of HAP were VAP in our study and excluding non-ventilated patients did not alter the observed results.

## Conclusions

*S. maltophilia* HAP had a very low incidence in critically ill patients but was associated with high mortality rate in this large multicenter study. Its onset is hard to predict because of lack of specific risk factors but occurs mainly in long-stay ICU patients. The present study did not provide evidence of a significant effect of delay, duration, or combination of antimicrobial therapy on mortality. Efforts in developing novel and effective approaches for prevention are warranted.

## Supplementary information

**Additional file 1.** **List of participating centers, collaborators and** **case-mix** **of ICU patients**.

**Additional file 2: Table S1.** **Invasive devices inserted at the diagnosis of** ***Stenotrophomonas maltophilia*** **hospital-acquired** **pneumonia**. Description of invasive devices inserted at the diagnosis of *Stenotrophomonas maltophilia* hospital-acquired pneumonia.

**Additional file 3: Table S2.** **Diagnosis methods for isolation of** ***Stenotrophomonas maltophilia.*** Description of diagnosis methods for isolation of *Stenotrophomonas maltophilia.*

**Additional file 4: Table S3.** **Treatment failure of** ***Stenotrophomonas maltophilia*** **hospital-acquired** **pneumonia**. Description of treatment failures of *Stenotrophomonas maltophilia* hospital-acquired pneumonia.

**Additional file 5: Table S4.** **Variables associated with the time to** **in-hospital** **death in patients with** ***S. maltophilia*** **ventilator-associated** **pneumonia**. Variables associated with the time to in-hospital death in patients with *S. maltophilia* ventilator-associated pneumonia.

**Additional file 6: Table S5.** **Propensity Score Matching**. Time to in-hospital death was compared between matched groups using a Cox proportional hazard model.

**Additional file 7: Table S6.** **Variable associated with** **time-to-death** **in the propensity matched population**. Variable associated with time-to-death in the propensity matched population.

## Data Availability

The datasets analyzed in this study are not publicly available due to privacy issues, but are available from the corresponding author upon reasonable request.

## Abbreviations

ARDS: Acute respiratory distress syndrome
AST: Antimicrobial susceptibility testing
BAL: Bronchoalveolar lavage
CFU: Colony-forming units
ETA: Endotracheal aspirate
HAP: Hospital-acquired pneumonia
ICU: Intensive care unit
MDR: Multidrug resistant
PSB: Protected specimen brush
PTC: Protected (plugged) telescoping catheter
SAPS: Simplified Acute Physiology Score
SOFA: Sequential organ failure assessment
TMP-SMX: Trimethoprim–sulfamethoxazole
VAP: Ventilator-associated pneumonia
