# Supplementary material for: Outcomes of Stenotrophomonas maltophilia hospital-acquired pneumonia in intensive care unit: a nationwide retrospective study
Source: Crit Care. 2019 Nov 21;23:371. doi: 10.1186/s13054-019-2649-5 (PMC6873544; doi:10.1186/s13054-019-2649-5)
Supplement: Supplementary file 1 — Additional file 1. List of participating centers, collaborators and case-mix of ICU patients. [file 13054_2019_2649_MOESM1_ESM.docx]

# Additional file 1: List of participating centers, collaborators and case-mix of ICU patients

**Dr Adrien Bouglé, MD. PhD.:** Department of Anesthesiology and Critical Care Medicine, Institute of Cardiology, Pitié-Salpêtrière Hospital, AP-HP, Paris, France; **Cardiothoracic surgical ICU**

**Dr Claire Charpentier MD.**: Réanimation chirurgicale Polyvalente, Centre Hospitalier Universitaire de Nancy, Nancy, France; **Surgical and** **Neurotrauma ICU**

**Prof. Olivier Collange MD. PhD.:** Département d’Anesthésie-Réanimation, Nouvel Hôpital Civil, Centre Hospitalier Universitaire, Strasbourg, France ; **Mixed ICU**

**Dr Fabrice Cook MD.:** Service d’Anesthésie-Réanimation, CHU Henri Mondor, Assistance Publique des Hôpitaux de Paris (AP-HP) ; **Neurotrauma ICU**

**Dr Nicolas Deye  MD. PhD.:** Medical & Toxicological Intensive Care Unit, Lariboisiere University Hospital, Assistance Publique des Hôpitaux de Paris (AP-HP), Paris ; **medical and toxicological ICU**

**Dr Clément Dubost MD.:** Service d’Anesthésie-Réanimation, Hôpital d’Instruction des Armées (HIA) Bégin, Saint-Mandé, France ; **mixed ICU**

**Dr Arnaud Foucrier MD. :** Service d’Anesthésie-Réanimation, Hôpital Beaujon, Assistance Publique des Hôpitaux de Paris (AP-HP), Clichy, France**; mixed surgical ICU**

**Dr Julie Gaudefroy MD.:** Département d’Anesthesie-Réanimation, Centre Hospitalier Universitaire Hautepierre, Strasbourg, France; **Surgical and** **Neurotrauma ICU**

**Dr Antoine Kimmoun MD. PhD.:** Department of Intensive Care Medicine, University Hospital of Nancy, Vandoeuvre-Lès-Nancy, France; **medical ICU**

**Dr Jean-Marc Lalot MD.:** Department of Anesthesiology and Critical Care Medicine, Hospital Emile Durkheim, Epinal, France; **mixed ICU**

**Dr Guillaume Louis MD.:** Department of Intensive Care Medicine, Hospital of Metz-Mercy, Metz, France; **mixed ICU**

**Dr Mathieu Mattei MD.:** Department of Anesthesiology and Critical Care Medicine, University Hospital of Nancy, Vandoeuvre-Lès-Nancy, France; **Cardiac surgical ICU**

**Prof. Armand Mekontso-Dessap MD. PhD. :** Service de Réanimation Médicale, CHU Henri Mondor, Assistance Publique des Hôpitaux de Paris (AP-HP) ; **Medical ICU**

**Dr Jean-Claude Merle MD.:** Service d’Anesthésie-Réanimation, CHU Henri Mondor, Assistance Publique des Hôpitaux de Paris (AP-HP) ; **Visceral and hepatobiliary surgical ICU**

**Prof. Paul-Michel Mertes MD. PhD.**: Département d’Anesthésie-Réanimation, Nouvel Hôpital Civil, Centre Hospitalier Universitaire, Strasbourg, France ; **Cardiovascular surgical ICU**

**Dr Nicolas Mongardon MD. PhD.:** Service d’Anesthésie-Réanimation, CHU Henri Mondor, Assistance Publique des Hôpitaux de Paris (AP-HP) ; **Cardiovascular surgical ICU**

**Prof. Romain Pirrachio MD. PhD.:** Department of Anesthesiology and Critical Care Medicine, University Hospital Georges Pompidou, AP-HP, Paris, France: **mixed surgical and trauma ICU**

**Prof. Julien Pottecher MD. PhD.:** Département d’Anesthesie-Réanimation, Centre Hospitalier Universitaire Hautepierre, Strasbourg, France; **Surgical and** **Neurotrauma ICU**

**Prof. Francis Schneider MD. PhD.:** Service de Réanimation Médicale, University Hospital of Hautepierre, Strasbourg, France; **Medical ICU**

**Dr Parvine Tashk MD.:** Hôpital Bichat-Claude-Bernard, Assistance Publique des Hôpitaux de Paris (AP-HP), Paris, France ; **mixed surgical ICU**

**From the AZUREA Research Network:**

**Prof Jean-Michel Constantin MD. PhD:** Pôle de Médecine Péri-Opératoire (MPO), Centre Hospitalier Universitaire de  Clermont-Ferrand, Clermont-Ferrand, France ; **mixed ICU**

**Dr Thomas Godet MD.:** Réanimation Adultes et Soins Continus, Pôle de Médecine Péri-opératoire, Hôpital Estaing, CHU de Clermont-Ferrand, France ; **mixed ICU**

**Dr Philippe Guerci MD.:** Department of Anesthesiology and Critical Care Medicine, Institut Lorrain du Coeur et des Vaisseaux, University Hospital of Nancy, Vandoeuvre-Lès-Nancy, France; **mixed** **surgical ICU**

**Dr Sebastien Perbet MD. PhD.:** Réanimation Médico-Chirurgicale, Pôle de Médecine Péri-opératoire, Hôpital Gabriel Montpied, CHU de Clermont-Ferrand, France ; **mixed ICU**

**Dr Stanislas Ledochowski MD.:** Service de Réanimation Polyvalente, Groupement Hospitalier Nord Dauphiné- Centre Hospitalier Pierre Oudot, Bourgoin-Jallieu, France ; **mixed ICU**
