# Supplementary material for: Outcomes of Stenotrophomonas maltophilia hospital-acquired pneumonia in intensive care unit: a nationwide retrospective study
Source: Crit Care. 2019 Nov 21;23:371. doi: 10.1186/s13054-019-2649-5 (PMC6873544; doi:10.1186/s13054-019-2649-5)
Supplement: Supplementary file 2 — Additional file 2: Table S1. Invasive devices inserted at the diagnosis of Stenotrophomonas maltophilia hospital-acquired pneumonia. Description of invasive devices inserted at the diagnosis of Stenotrophomonas maltophilia hospital-acquired pneumonia. [file 13054_2019_2649_MOESM2_ESM.docx]

# Additional table S1: Invasive devices inserted at the diagnosis of *Stenotrophomonas maltophilia* hospital-acquired pneumonia

| **Variables** | **Total**  **N=282** |
| --- | --- |
| Arterial line | 262 (92.9) |
| Central venous catheter | 259 (91.8) |
| Dialysis catheter | 72 (25.5) |
| Tracheostomy | 36 (12.8) |
| Veno-venous ECMO | 4 (1.4) |
| Veno-arterial ECMO | 13 (4.6) |

Number of devices inserted (n, (%)) considering the whole cohort of 282 patients

ECMO: Extracorporeal Membrane Oxygenation
