# Supplementary material for: Outcomes of Stenotrophomonas maltophilia hospital-acquired pneumonia in intensive care unit: a nationwide retrospective study
Source: Crit Care. 2019 Nov 21;23:371. doi: 10.1186/s13054-019-2649-5 (PMC6873544; doi:10.1186/s13054-019-2649-5)
Supplement: Supplementary file 3 — Additional file 3: Table S2. Diagnosis methods for isolation of Stenotrophomonas maltophilia. Description of diagnosis methods for isolation of Stenotrophomonas maltophilia. [file 13054_2019_2649_MOESM3_ESM.docx]

# Additional table S2: Diagnosis methods for isolation of *Stenotrophomonas maltophilia*

| **Variables** | **Total**  **N=281** |
| --- | --- |
| Sputum cytologic examination | 10 (3.6) |
| Tracheobronchial aspiration | 95 (33.8) |
| Protected specimen brush or protected (plugged) telescoping catheter | 103 (36.6) |
| Broncho-alveolar lavage | 73 (25.9) |
