# Supplementary material for: Outcomes of Stenotrophomonas maltophilia hospital-acquired pneumonia in intensive care unit: a nationwide retrospective study
Source: Crit Care. 2019 Nov 21;23:371. doi: 10.1186/s13054-019-2649-5 (PMC6873544; doi:10.1186/s13054-019-2649-5)
Supplement: Supplementary file 4 — Additional file 4: Table S3. Treatment failure of Stenotrophomonas maltophilia hospital-acquired pneumonia. Description of treatment failures of Stenotrophomonas maltophilia hospital-acquired pneumonia. [file 13054_2019_2649_MOESM4_ESM.docx]

# Additional table S3: Treatment failure of *Stenotrophomonas maltophilia* hospital-acquired pneumonia

| **Variables** | **Total**  **N=282** |
| --- | --- |
| Failure of first line treatment or death attributed to *S. maltophilia* HAP | 65 (23.1) |
| Recurrence of pneumonia documented at *S. maltophilia* | 48 (17.0) |
| New onset of pneumonia documented at another bacteria | 70 (24.8) |
| **Other microorganism documented in new pneumonia** |  |
| *Acinetobacter baumanii* | 3 (4.3) |
| *Citrobacter spp* | 2 (2.9) |
| *Enterobacter spp* | 9 (12.9) |
| *Enterococcus spp* | 7 (10) |
| *Escherichia coli* | 3 (4.3) |
| *Klebsiella spp* | 17 (24.3) |
| Methicillin-resistant *Staphylococcus aureus* | 2 (2.9) |
| Methicillin-sensitive *Staphylococcus aureus* | 2 (2.9) |
| *Morganella morganii* | 1 (1.5) |
| *Proteus spp* | 2 (2.9) |
| *Pseudomonas aeruginosa* | 30 (42.9) |
| *Serratia spp* | 1 (1.5) |
| *Streptococcus spp* | 1 (1.5) |

Data are expressed as number and percentage. HAP: Hospital acquired-pneumonia
