# Supplementary material for: Outcomes of Stenotrophomonas maltophilia hospital-acquired pneumonia in intensive care unit: a nationwide retrospective study
Source: Crit Care. 2019 Nov 21;23:371. doi: 10.1186/s13054-019-2649-5 (PMC6873544; doi:10.1186/s13054-019-2649-5)
Supplement: Supplementary file 5 — Additional file 5: Table S4. Variables associated with the time to in-hospital death in patients with S. maltophilia ventilator-associated pneumonia. Variables associated with the time to in-hospital death in patients with S. maltophilia ventilator-associated pneumonia. [file 13054_2019_2649_MOESM5_ESM.docx]

# Additional table S4: Variables associated with the time to in-hospital death in patients with *S. maltophilia* ventilator-associated pneumonia

| **Variables** | **Univariate analysis** | | **Multivariate analysis** | |
| --- | --- | --- | --- | --- |
|  | **HR [IC 95%]** | **p-value** | **HR [IC 95%]** | **p-value** |
| Age | 1.026 [1.011; 1.041] | 0.0006 | 1.02 [1.01; 1.04] | 0.003 |
| SAPS II | 1.008 [0.999 ; 1.018] | 0.0925 |  |  |
| Mechanical ventilation at diagnosis | 1.341 [0.55 ; 3.29] | 0.5223 |  |  |
| Duration of MV before the diagnosis | 0.999 [0.992 ; 1.005] | 0.7423 |  |  |
| SOFA score at diagnosis | 1.096 [1.050 ; 1.144] | <0.0001 | 1.1 [1.05; 1.15] | <0.001 |
| Bacteremia | 0.899 [0.42 ; 1.94] | 0.7856 |  |  |
| Monomicrobial pneumonia | 1.302 [0.90 ; 1.89] | 0.1675 |  |  |
| Co-infection with *Pseudomonas aeruginosa* | 0.887 [0.55 ; 1.44] | 0.6279 |  |  |
| Empirical antibiotic therapy | 0.828 [0.57 ; 1.20] | 0.3208 |  |  |
| Empirical antibiotic therapy effective against *S. maltophilia* | 0.951 [0.56 ; 1.61] | 0.8532 |  |  |
| Effective combination antimicrobial therapy | 1.25 [0.83; 1.88] | 0.295 |  |  |
| Duration of effective antimicrobial therapy against *S. maltophilia* | 1.03 [0.98;1.08] | 0.198 |  |  |
| Duration of effective combination therapy against *S. maltophilia* | 0.84 [0.52; 1.35] | 0.463 |  |  |

SAPS: Simplified Acute Physiology Score, MV: mechanical ventilation, HR: hazard ratio, *S. maltophilia*: *Stenotrophomonas maltophilia*, SOFA: Sequential Organ Failure Assessment
