# Supplementary material for: Outcomes of Stenotrophomonas maltophilia hospital-acquired pneumonia in intensive care unit: a nationwide retrospective study
Source: Crit Care. 2019 Nov 21;23:371. doi: 10.1186/s13054-019-2649-5 (PMC6873544; doi:10.1186/s13054-019-2649-5)
Supplement: Supplementary file 6 — Additional file 6: Table S5. Propensity Score Matching. Time to in-hospital death was compared between matched groups using a Cox proportional hazard model. [file 13054_2019_2649_MOESM6_ESM.docx]

# Additional table S5: Propensity Score Matching

| **Variable** | **Naive** | **Weighted** | **No EAT (n or mean)** | **No EAT (SD or prop)** | **ETA**  **(n or mean)** | **EAT**  **(SD or prop)** | **Weighted no EAT (n or mean)** | **Weighted no EAT (SD or prop)** | **Weighted EAT**  **(n or mean)** | **Weighted EAT**  **(SD or prop)** |
| --- | --- | --- | --- | --- | --- | --- | --- | --- | --- | --- |
| Age | 0.061 | 0.098 | 63.771 | 16.561 | 62.250 | 14.342 | 63.771 | 16.561 | 62.250 | 14.342 |
| Duration of ICU stay before diagnosis | 0.102 | 0.064 | 18.667 | 18.758 | 17.583 | 14.911 | 18.667 | 18.758 | 17.583 | 14.911 |
| SAPS 2 | 0.131 | 0.038 | 45.083 | 18.342 | 45.812 | 20.478 | 45.083 | 18.342 | 45.812 | 20.478 |
| SOFA | 0.046 | 0.068 | 8.188 | 3.779 | 7.896 | 4.692 | 8.188 | 3.779 | 7.896 | 4.692 |
| Female | 0.058 | 0.046 | 14.000 | 0.292 | 13.000 | 0.271 | 14.000 | 0.292 | 13.000 | 0.271 |
| Male | 0.068 | 0.046 | 34.000 | 0.708 | 35.000 | 0.729 | 34.000 | 0.708 | 35.000 | 0.729 |

EAT: Empirical antibiotic therapy effective against *S. maltophilia*
