# Supplementary material for: Outcomes of Stenotrophomonas maltophilia hospital-acquired pneumonia in intensive care unit: a nationwide retrospective study
Source: Crit Care. 2019 Nov 21;23:371. doi: 10.1186/s13054-019-2649-5 (PMC6873544; doi:10.1186/s13054-019-2649-5)
Supplement: Supplementary file 7 — Additional file 7: Table S6. Variable associated with time-to-death in the propensity matched population. Variable associated with time-to-death in the propensity matched population. [file 13054_2019_2649_MOESM7_ESM.docx]

**Additional table S6: Variable associated with time-to-death in the propensity matched population**

| **Variable** | **Hazard Ratio** | **Confidence interval 95%** | **P value** |
| --- | --- | --- | --- |
| Empirical antibiotic therapy effective against *S. maltophilia* | 0.891 | [0.498 – 1.593] | 0.697 |
